# The effect of different nasal irrigation solutions following septoplasty and concha radiofrequency: a prospective randomized study^☆^

**DOI:** 10.1016/j.bjorl.2017.01.005

**Published:** 2017-02-22

**Authors:** Hanifi Kurtaran, K. Serife Ugur, Ceyda Sel Yilmaz, Mesut Kaya, Alper Yuksel, Nebil Ark, Mehmet Gunduz

**Affiliations:** Turgut Ozal University Faculty of Medicine, Department of Otorhinolaryngology Head and Neck Surgery, Ankara, Turkey

**Keywords:** Septoplasty, Nasal irrigation, Mucociliary clearance, Crusting, Obstruction, Septoplastia, Irrigação nasal, *Clearance* mucociliar, Crostas, Obstrução

## Abstract

**Introduction:**

Nasal irrigation solutions are widely used following endonasal surgery. These irrigation solutions remove infective debris and crusts, reducing the probability of synechia formation, and accelerate mucosal healing.

**Objective:**

The aim of the present study was to compare the effects of nasal irrigation solutions with different contents following septoplasty and concha radiofrequency.

**Methods:**

The present study was a prospective, randomized, controlled simple blind study of 120 patients who underwent septoplasty and bilateral concha radiofrequency. Patients were divided into four groups according to the nasal irrigation solution used: tap water, buffered isotonic saline, saline with xylitol, and hypertonic sea water. Patients were examined on the 7th and 15th postoperative days. A saccharine test was applied to determine mucociliary activity preoperatively and on the 7th and 15th postoperative days. Patients were asked about drying and obstruction using a 10 cm visual analog scale. In addition, patients were examined to determine the crusting score.

**Results:**

There was no significant difference found in the preoperative and 7th and 15th postoperative days’ mucociliary clearance times among the four groups. The crusting score was found to be significantly lower in the hypertonic sea water group (*p* < 0.001). Drying and obstruction on the 7th and 15th postoperative days were found to be significantly more comfortable in the hypertonic sea water group (*p* < 0.001).

**Conclusion:**

Hypertonic sea water is the recommended irrigation solution, as it is associated with less crusting, drying, and obstruction in the nose for the postoperative period following septoplasty and concha radiofrequency.

## Resumo

**Introdução:**

Soluções para irrigação nasal são amplamente utilizadas após cirurgias endonasais. Essas soluções removem os resíduos e crostas, reduzindo a probabilidade de formação de sinéquias e acelerando a cicatrização da mucosa.

**Objetivo:**

O objetivo do presente estudo foi comparar os efeitos das soluções para irrigação nasal com diferentes conteúdos após septoplastia e turbinoplastia com radiofrequência.

**Método:**

O presente estudo foi um estudo cego simples, randomizado, controlado e prospectivo de 120 pacientes submetidos à septoplastia e turbinoplastia bilateral com radiofrequência. Os pacientes foram divididos em quatro grupos de acordo com a solução nasal utilizada: água da torneira, solução salina isotônica tamponada, solução salina com xilitol e água do mar hipertônica. Os pacientes foram examinados no 7° e 15° dias do pós-operatório. O teste de sacarina foi utilizado para determinar a atividade mucociliar pré-operatória e no 7° e 15° dias do pós-operatório. Os pacientes foram questionados sobre a sensação de secura e obstrução nasais utilizando uma escala visual analógica de 10 cm. Além disso, os pacientes foram examinados para determinar o escore em relação à crostas.

**Resultados:**

Não houve diferença significativa entre o pré-operatório e os 7° e 15° dias do pós-operatório dos tempos de *clearance* mucociliar entre os quatro grupos. Verificou-se que o escore em relação a crostas foi significativamente menor no grupo utilizando água do mar hipertônica (*p* < 0,001). As sensações de secura e obstrução nasais no 7° e 15° dias do pós-operatório mostraram-se significativamente mais confortáveis no grupo água do mar hipertônica (*p* < 0,001).

**Conclusão:**

A água de mar hipertônica é a solução de irrigação recomendada, pois está associada a menor incidência de crostas, secura e obstrução nasais no pós-operatório de cirurgia de septoplastia e das conchas nasais com radiofrequência.

## Introduction

Nasal irrigation solutions are widely used following endonasal surgery and are usually a part of rhinitis and sinusitis treatment. These irrigation solutions remove infective debris and crusts, reducing the probability of synechia formation, and they accelerate mucosal healing.^1^ At the same time, they improve mucociliary clearance.^2^

Solutions with different contents are currently used for nasal irrigation. Isotonic saline solution has been the most preferred one for a long time^3^; however, more recently the use of hypertonic saline has increased. Studies show that hypertonic solutions reduce edema, improve mucociliary clearance, and relieve nasal breathing by affecting osmotic pressure.^1^, ^2^, ^4^ However, Homer et al.^5^ found that 3% hypertonic saline and isotonic saline solutions do not differ in their effects on mucociliary clearance. Furthermore, Suslu et al.^6^ showed that hypertonic saline solutions are significantly more effective on mucociliary clearance then isotonic solutions, with improvement in nasal airway dimensions. However, the use of hypertonic solutions is restricted due to side effects including nasal irritation and burning sensation.^2^

This study compared the effects of four irrigation solutions on mucociliary clearance, nasal crusting, nasal dryness, and obstruction following septoplasty and bilateral concha radiofrequency. Many studies have been conducted on the effects of these solutions as used for treatment of rhinitis, sinusitis, and septoplasty; however, a search revealed no studies on their effects when used to treat septoplasty and concha radiofrequency. The current study used tap water, buffered isotonic saline, buffered isotonic saline with xylitol, and hypertonic sea water for irrigation. This is the first study comparing tap water to other solutions, which is noteworthy, as tap water is an attractive option in developing countries.

## Methods

A prospective, simple blind, randomized study was conducted on 120 volunteers undergoing septoplasty and concha radiofrequency in the period between December 2012 and December 2014. Informed consent was obtained from all subjects and the study was approved by the Institutional Review Board (IRB 270).

The operations were performed in all four seasons in a city with a continental climate.

The same surgical procedures and techniques were performed on all subjects. The surgeries were done by different surgeon. The examination of the patients were performed by same otolaryngologist. A standard hemitransfiction incision was first made along the edge of the septal cartilage, followed by mucoperichondrial and mucoperiosteal flap elevation. The deviated portions of the cartilaginous septum and bony septum were removed (leaving at least 1 cm of dorsal and anterior cartilage for support) to obtain a straight septum.

The RF generator (Gyrus ENT, USA) was set to deliver 300 J, with a target temperature of 75 °C. Energy was delivered by inserting the active portion of the needle electrode longitudinally into the submucosa of the anterior and middle parts of the inferior conchae of both sides.

Postoperative Doyle Nasal Splints (Boston medical products, MA, USA) were placed during the procedures and removed on the 2nd postoperative day. All patients received the same postoperative medication: Amoxicillin/clavulanate for 7 days and paracetamol.

Subjects were randomly divided into 4 groups and one of the 4 nasal irrigation solutions was administered to each group. Both the patients and the investigators were blinded to which solution was administered. The groups each comprised 30 patients. Group 1 was administered tap water; Group 2 was given Buffered Isotonic Saline (BIS) (Neilmed^®^ Sinus Rinse™, NaCl 10 mg/mL, NaHCO_3_ 0.5–1 mg/mL); Group 3 was given Buffered Isotonic Saline with xylitol (BISX) (Entrelief^®^ Rahat Nefes, 3.5 gr); and Group 4 was administered Hypertonic Sea Water (HSW) (Sinomarin^®^ ENT, 2.3% NaCl).

All subjects were instructed to use irrigation fluids 3 times a day for a period of 15 days following the removal of the nasal splints. A total of 60 mL of solution was used for each nasal irrigation, with 30 mL for each nasal cavity. Tap water was performed using a syringe.

A saccharine clearance test method was used to measure Mucociliary Clearance Time (MCT) in every patient preoperatively and also on 7th and 15th day postoperatively. A quarter of a saccharin tablet was placed just beneath the anterior end of the inferior concha. Patients sat in an upright position and were asked to avoid sniffling and sneezing. The time which passed before the patient could taste the saccharine was recorded as MCT.

Crusting in the nasal cavity was evaluated on the 7th and 15th days postoperatively by a 0° nasal endoscope. Crusting scores were set as follows: 0 (none), 1 (mild), 2 (moderate), and 3 (severe). Nasal dryness and obstruction were evaluated using a 10 cm visual analog scale, with lack of dryness and obstruction at the right end of the scale (10 cm) and the worst symptoms at the left end of the scale (0 cm). The groups’ visual analog scale scores were compared.

Exclusion criteria were upper airway infection within six weeks prior to surgery, systemic disease, rhinitis, smoking, and topical nasal spray use within three weeks prior to surgery.

### Statistical methods

The Statistical Package Program for the Social Sciences (SPSS 15.0; SPSS Inc., Chicago, IL, USA) was used for statistical analysis. The Shapiro–Wilk test was used to ensure distributional adequacy. Numbers and percentages were used to present categorical data and a Chi Square was used for comparison. A median (minimum and maximum) was used for continuous variables. The Kruskal–Wallis test and the Mann–Whitney *U* test with Bonferroni correction were used to compare independent groups. The Friedman test was used to compare dependent samples and the Wilcoxon test was used when comparing two related samples. The statistical significance was set as *p* < 0.05.

## Results

The study population included 120 adult patients. The median age of the patients was 32.5 (17–64). The median ages in Groups 1 through 4 were 34 (18–64), 30 (17–46), 34 (18–63) and 33 (22–64), respectively. There were no significant differences in age distribution among the groups (*p* = 0.432).

The study sample consisted of 81 (67.5%) males and 39 (32.5%) females. There were no significant differences in gender distribution among the groups (*p* = 0.415).

Each group was examined separately. In Groups 1 and 4, MCT values were not significantly changed on the 7th postoperative day as compared to preoperative measurements (*p* > 0.05). However, on the 15th postoperative day, the MCT values of these two groups were significantly shorter as compared to the preoperative and 7th postoperative day values (*p* < 0.05). In Group 2, the MCT values improved significantly from the preoperative measurements to the 7th postoperative day measurements as well as from the 7th postoperative day to the 15th postoperative day (*p* < 0.05). In Group 3, there were no significant changes found on the 7th and 15th postoperative days (*p* > 0.05) (Table 1).

**Table 1:** Effects of nasal irrigation solutions on MCT.

	Group 1		Group 2		Group 3		Group 4	
MCT	(*n* = 30)	*p*	(*n* = 30)	*p*	(*n* = 30)	*p*	(*n* = 30)	*p*
*Preop – 7th day*								
Negative	18	0.351	21	0.014	21	0.152	18	0.151
Positive	11		8		8		9	
Ties	1		1		1		3	

*Preop – 15th day*								
Negative	24	0.001	22	0.001	19	0.135	18	0.020
Positive	6		7		10		6	
Ties	0		1		1		6	

*7th – 15th day*								
Negative	23	0.001	20	0.047	18	0.359	17	0.028
Positive	4		7		9		9	
Ties	3		3		3		4	

Statistical significance: set as *p* < 0.05.

When the groups were compared, no significant differences were found in the preoperative or 7th and 15th postoperative day MCT scores among the 4 groups (*p* = 0.377, *p* = 0.386, *p* = 0.521 respectively).

Each in-group variation in MCT scores was compared with the other groups’ score variations, and no significant differences were found (7th postoperative day–preoperative, *p* = 0.364; 15th postoperative day–preoperative, *p* = 0.316; 15th postoperative day–7th postoperative day, *p* = 0.242).

Crusting score changes were not significant in Groups 1 and 3 (*p* = 0.655 and *p* = 0.132, respectively) on the 7th and 15th postoperative days. However, in Groups 2 and 4, significant changes were found (*p* < 0.05) (Table 2).

**Table 2:** Crusting score comparison.

	Group 1	Group 2	Group 3	Group 4	*p*
Postoperative 7th day	1 (1–3)	2 (0–3)	1 (0–3)	1 (0–2)	<0.001^a^
Postoperative15th day	1 (1–3)	1 (0–3)	1 (0–3)	1 (0–1)	<0.001^a^

a Group 4 was significantly lower than others. Statistical significance: set as *p* < 0.05.

The groups’ crusting scores from the 7th and 15th postoperative days were compared to each other. Group 4's scores were significantly lower than the others (*p* < 0.001). There were no significant differences among the other groups (*p* > 0.05).

Each in-group variation for crusting was compared with other groups’ crusting measurement variation, and no significant differences were found (*p* = 0.294).

When in-group nasal obstruction scores were analyzed, the 15th postoperative day scores showed significant improvement compared to the 7th postoperative day scores in Groups 1 and 4 (*p* = 0.022 and *p* = 0.005). However, nasal dryness was significantly improved in Group 2 (*p* = 0.007). There were no significant changes found in obstruction or dryness in the other groups (*p* > 0.05) (Table 3).

**Table 3:** Distribution of VAS scores changes in groups for dryness and obstruction from 7th to 15th postoperative day.

	Group 1		Group 2		Group 3		Group 4	
VAS	(*n* = 30)	*p*	(*n* = 30)	*p*	(*n* = 30)	*p*	(*n* = 30)	*p*
*Dryness*								
Negative	15	0.991	18	0.007	13	0.666	11	0.896
Positive	13		6		12		15	
Ties	2		6		5		4	

*Obstruction*								
Negative	7	0.022	15	0.783	11	0.934	3	0.005
Positive	20		9		14		17	
Ties	3		6		5		10	

Statistical significance: set as *p* < 0.05.

The groups’ dryness and obstruction scores on the 7th and 15th postoperative days were compared to each other. Group 4's VAS scores were significantly higher than the other groups’ (*p* < 0.001). Dryness on the 15th day and obstruction on the 7th day VAS scores were significantly higher in Group 2 as compared to Groups 1 and 3 (respectively, *p* = 0.005 and *p* = 0.049). Dryness VAS scores in Group 3 were significantly higher than in Group 1 (respectively, *p* = 0.019 and *p* = 0.001) (Table 4).

**Table 4:** Comparison of groups for VAS scores of dryness and obstruction.

	Group 1	Group 2	Group 3	Group 4	*p*
Dryness 7th day	5 (2–10)	7 (3–10)	8 (1–10)	8.5 (7–10)	0.019^b^; <0.001^c^
Dryness 15th day	6 (2–9)	5 (2–10)	8 (2–10)	8.5 (5–10)	0.001^b^; 0.005^a^
Obstruction 7th day	5 (0–8)	6 (2–10)	5.5 (2–10)	8 (5–10)	0.049^a^; <0.001^c^
Obstruction 15th day	5.5 (2–9)	6 (1–10)	6 (2–10)	9 (5–10)	<0.001^c^

a Difference in group 2 with groups 1 and 3.

b Difference in group 1 and group 3.

c Differences in group 4 with others. Statistical significance: set as *p* < 0.05.

Each in-group variation for dryness and crusting was compared with other groups, and no significant difference was found (respectively *p* = 0.126 and *p* = 0.059).

## Discussion

Nasal irrigation solutions are widely used for sinusitis treatment and for removing nasal secretions, infective debris and crusts following nasal surgery. Irrigation solutions contain different minerals and chemicals. Many studies have been conducted using different topical nasal solutions with varying results.^1^, ^5^, ^6^, ^7^, ^8^, ^9^ The present study used four solutions and compared their effects on patients following septoplasty and bilateral concha radiofrequency.

Mucociliary clearance has an important role in nasal defense mechanisms following endonasal surgery. The saccharine clearance test is the preferred method for measuring mucociliary clearance because it is easily applied, inexpensive, and reliable.^7^ As is well known, endonasal surgery has adverse effects on mucociliary clearance. Irrigation solutions are employed to reduce these adverse effects as part of postoperative treatment.^9^ Tap water and isotonic (not buffered) saline solutions are easy to available and widely used in the our country. Recently, the use of hypertonic saline has steadily increased because it reduces edema and increases mucociliary clearance.^4^, ^5^ Talbot et al.,^1^ Keojampa et al.,^2^ and Suslu et al.^6^ have reported that hypertonic saline increases mucociliary clearance, whereas Homer et al.^5^ and Low et al.^10^ reported no difference between isotonic saline and hypertonic saline in terms of mucociliary clearance. The present study revealed no significant difference in the preoperative and both postoperative MCT scores for Group 3 (BISX), but significant differences were found in all the other groups. There were no significant differences in MCT scores among the 4 groups.

Nasal irrigation solutions clear the nose and provide more comfortable breathing. It is said that hypertonic solutions cause nasal tenderness and irritation,^11^, ^12^ where others say osmotic pressure, reduce mucosal edema, and ameliorate breathing.^1^ Keojampa et al.^2^ found no difference between isotonic and hypertonic salines’ effect on breathing, whereas Hauptman et al.^13^ found that buffered isotonic saline significantly relieves nasal obstruction as compared to hypertonic saline and Suslu et al.^6^ reported that buffered hypertonic saline relieves nasal obstruction better than non-buffered isotonic saline irrigation solutions. The present study compared the obstruction VAS values on the 7th and 15th postoperative days, finding that the HSW group's values were significantly high (*p* < 0.001). Furthermore, the HSW group's crusting score on the 7th and 15th postoperative days were significantly low (*p* < 0.001). There were no significant differences among the other groups (*p* > 0.05).

Some studies note that patients avoid the use of hypertonic saline due to the nasal irritation it inflicts.^1^, ^11^ Hauptman et al.^13^ reported that buffered hypertonic solutions induce nasal irritation and cause burning sensation more than buffered isotonic solutions. Suslu et al.^6^ reported that buffered isotonic saline causes less burning sensation than non-buffered hypertonic and non-buffered isotonic saline and that the acidity of the solution is an important factor in burning sensation. Salib et al. reported that high-volume, low-pressure Sterimar™ saline irrigations are more effective than low-volume, high-pressure Sinus Rinse™ saline irrigations following endoscopic sinus surgery in the early postoperative period; however, there were no differences noted in ease of use.^14^ The present study evaluated the dryness VAS values on the 7th and 15th postoperative days and found the HSW group's values to be significantly high. These findings may have been caused by the concentration of the hypertonic solution; the present study used a 2.3% solution, whereas others^1^, ^2^, ^13^ used a 3% solution. The use of HSW improves edema and crusting of concha following radiofrequency.

Tap water nasal irrigation is widely used in developing countries following endonasal surgery. Use of tap water in treatments of sinusitis and seasonal allergic rhinitis has been studied, but not its use following endonasal surgery.^15^, ^16^ The present study found that tap water is less effective than other solutions for relieving dryness and obstruction. In addition, tap water poses an extremely low risk of meningoencephalitis from Naegleria fowleri, which makes this a potential health hazard.

The limitations of this study are not double-blinded, lack of rhinomanometry, our scores were dependent on a subjective opinion of the patients and lack of measurements of the nasal irritation and burning sensation which restrict the use of hypertonic solutions.

## Conclusion

Hypertonic sea water, especially 2.3%, is the best solution for relieving nasal crusting, dryness, and obstruction following septoplasty and concha radiofrequency.

## Conflicts of interest

The authors declare no conflicts of interest.
